# Exploring the Potential of ^87^Sr/^86^Sr Isotope Ratio with Strontium and Rubidium Levels to Assess the Geographic Origin of Saffron

**DOI:** 10.3390/foods12152830

**Published:** 2023-07-26

**Authors:** Micha Horacek, Mounira Lage, Jyoti Vakhlu

**Affiliations:** 1 BLT Wieselburg, Rottenhauserstr. 1, 3250 Wieselburg, Austria; 2 Department of Lithospheric Research, Vienna University, 1090 Vienna, Austria; 3 National Institute of Agronomique Research (INRA), Rabat 10000, Morocco; 4 School of Biotechnology, University of Jammu, Jammu 18006, India; jyotivakhlu@jammuuniversity.ac.in

**Keywords:** provenance, spice, bedrock geology, MC-ICPMS, consumer deception, fraught, strontium isotope

## Abstract

Saffron is regarded as the most expensive spice, mainly because of its laborious harvest. Only a few countries dominate the global saffron market, with Iran producing by far the most saffron, and the saffron production of all other countries thus being much smaller. However, the respective national production (not only of saffron) is usually preferred by local consumers with respect to foreign products and often has a higher price. Cases of saffron with mislabeled geographic origin have repeatedly occurred. Thus, to protect local saffron production, control of the declared geographic origin is required. In the present case, differentiation of the geographic origin by ^87^Sr/^86^Sr is performed. The results show the saffron of several countries of origin to vary within the range of marine carbonates; however, saffron samples of Moroccan and Indian origin mainly show elevated ^87^Sr/^86^Sr values. Within the Indian saffron samples, one sample from Kishtwar Valley can be differentiated from the Kashmir saffron samples. The results are thus promising, especially when using the combination of Sr and Rb concentrations to differentiate geographic origin whenever the regions are of homogenous bedrock geology within and of different geology between the regions. However, the reported findings need to be checked and confirmed by further and additional saffron samples.

## 1. Introduction

Saffron is regarded as the most expensive spice in the world with respect to its weight [1]. This is mainly due to the laborious harvesting by hand, as 1 kg of saffron consists of ca. 250,000 crocus blossom stigmata [1]. Thus, since ancient times, saffron buyers risk being deceived by unscrupulous fraudsters who increase the weight of the saffron by the addition of substances, admix or even replace the saffron with other materials, and, more recently, incorrectly declare the geographic origin of the saffron. About 90% of all saffron produced globally originates from Iran. Iranian saffron is usually also cheaper than the saffron of other geographic origins. In many countries and regions, local and regional products are preferred to “alien” saffron, and substantially higher prices are paid for the local/regional product, especially in, but not restricted to, Europe. Therefore, there exists a relevant difference in price between the cheapest saffron available and the more expensive European (e.g., Greece, Italy, Spain, Austria, Switzerland, UK) saffron, and thus, there is the risk of consumer deception by incorrect declaration of the geographic origin of saffron. For example, there have been repeated claims of (Iranian) saffron being sold under the label of Spanish geographic origin (e.g., [2,3,4]). Thus, it seems there already exists a long history of incorrect declaration of the geographic origin of saffron and therefore consumer deception, at least concerning Spanish saffron. Furthermore, it needs to be kept in mind that this fraud, besides deceiving the consumer, also harms the honest saffron producers and traders in Spain, as they can be outcompeted with respect to the price of saffron. Finally, if Iranian saffron of lesser quality is declared to be of Spanish (or other esteemed) origin, this harms the consumer esteem for Spanish saffron and consequently the demand, and the willingness to pay a higher price for Spanish saffron and generally for saffron of certain geographic origins thus marketed at a higher price.

Conventionally, control of the correct declaration of geographic origin is carried out by control of the accompanying paperwork. However, it has been shown that this is insufficient, as goods can be (accidentally or intentionally) exchanged, mixed, replaced, etc., which cannot be detected by control of the accompanying documents and certificates. Therefore, control of the product itself is necessary. Usually, in food control, application of stable isotope analysis (SIA) of light elements (H, C, N, O, S) is the method of choice (e.g., [5,6,7,8,9,10,11,12,13,14], among many others). Maggi et al. (2011) [15] published the first study on the differentiation of saffron geographic origin by stable isotope analysis, and a second study was published by Wakefield et al. (2019) [16], differentiating Spanish and Iranian saffron samples using stable isotope ratios and elemental concentrations. Perini et al. (2021) [17] published a similar study differentiating Italian saffron from the saffron of other (Iranian, Moroccan) origins. However, often the control by light element SIA in comparison with reference samples requires authentic sample results from the same vintage to account for variations in the isotope ratios between years, owing to differing weather and agricultural practices (e.g., [5,6,12,17,18]). In this way the article by Wakefield et al. (2019) [16] is misleading, as it reports a good distinction between Iranian and Spanish saffron by SIA (δ^2^H and δ^13^C). However, concerning δ^13^C, this was only the case when they compared the combined results of Iranian saffron from 2010 and 2011 with the results of Spanish saffron from 2010. Comparison of only the 2010 vintage of Iranian and Spanish saffron revealed no significant difference in δ^13^C. For ^2^H, it seems that there was a problem with their Iranian ^2^H data, as they reported means of −80‰ for 2010 and −79‰ for 2011 but a mean of −67‰ for the combined 2010 and 2011 samples. Assuming that the Iranian values for the individual years 2010 and 2011 are correct, the mean for both years must be ca. −79.5‰. For the Spanish saffron they reported a mean of −75‰ but also a range from −97‰ to −75‰; hence, this mean also appears to be doubtful. Because of these inconsistencies, it seems that the data of Wakefield et al. (2019) [16] generally need to be regarded with caution.

Other investigations concerning the geographic origin of saffron include the analysis of components (such as safranal, crocin, picocrocin, etc.) in saffron [19,20,21,22,23,24,25,26], although Wakefield et al. (2019) [16] noted that they might be altered owing to different drying techniques and during storage. Another promising attempt was published by Sharma et al. (2012) [27] investigating the microbiome of saffron corms of different geographic origins.

The investigation of a geogenic parameter, which does not change between seasons and years/vintages, would facilitate such controls significantly, as reference material would not need to be of the same vintage. The isotope ratio of strontium is recognized as such a marker, as was demonstrated by Agguzzoni et al. (2020) [28] and Cellier et al. (2021) [29], and numerous publications reported its successful applications (e.g., [30,31,32]). This method describes and analyzes the transfer of ^87^Sr/^86^Sr from the soil and the bedrock into the growing plants (and, when applicable, from the plants into the animals feeding on them) without any relevant change in ^87^Sr/^86^Sr along the transfer (and food) chain. Generally, the type of bedrock, which by weathering and erosion transforms into the overlying soil, defines the ^87^Sr/^86^Sr-composition. Marine carbonates have a very specific ^87^Sr/^86^Sr range between 0.7068 and 0.7092 (e.g., [33], and references therein), basaltic rocks usually have ratios below that range, and siliciclastic rocks including acidic volcanics, granites, and gneiss usually have ratios beyond the range of the marine carbonates. Moreover, siliciclastic impurities in carbonates can result in values beyond the marine carbonate range.

Cellier et al. (2021) [29] demonstrated that the ^87^Sr/^86^Sr values in Champagne have a narrow range (within the marine carbonate range), and thus the ^87^Sr/^86^Sr values of sparkling wines from many other countries, even though they are overlapping with the Champagne range, are outside of this narrow interval. Epova et al. (2018) [34] documented the strong influence of salt in cured ham on its ^87^Sr/^86^Sr values, similar to the study of Tchaikovsky et al. (2019) [35] on salted caviar. A further advantage of the ^87^Sr/^86^Sr proxy is the fact that its isotope ratio is transferred into biogenic tissue without fractionation [36]. Thus, potentially, different types of materials can be used as references, as there is no need to compare the exact same type of sample and reference material (as is the case for the light element SIA).

However, some investigations have demonstrated the limitations (e.g., because of geological heterogeneities, difference in the ^87^Sr/^86^Sr of bedrock and covering sediment, changing of the natural ^87^Sr/^86^Sr owing to anthropogenic activities, etc.) of this method (e.g., [28,33,37,38,39]), and Horacek et al. (2022) [40] concluded that the value and power of this approach depends on the exact question that needs to be answered and the respective geological setting.

In the present study, the differentiation of saffron samples from various origins (most of them relevant saffron production countries/areas) by ^87^Sr/^86^Sr analysis is explored. To our knowledge, this is the first study authenticating the geographic origin of saffron using ^87^Sr/^86^Sr. We hypothesize that differentiation of the respective origins should be possible by analysis of the ^87^Sr/^86^Sr ratio.

## 2. Materials and Methods

**Figure 1:**
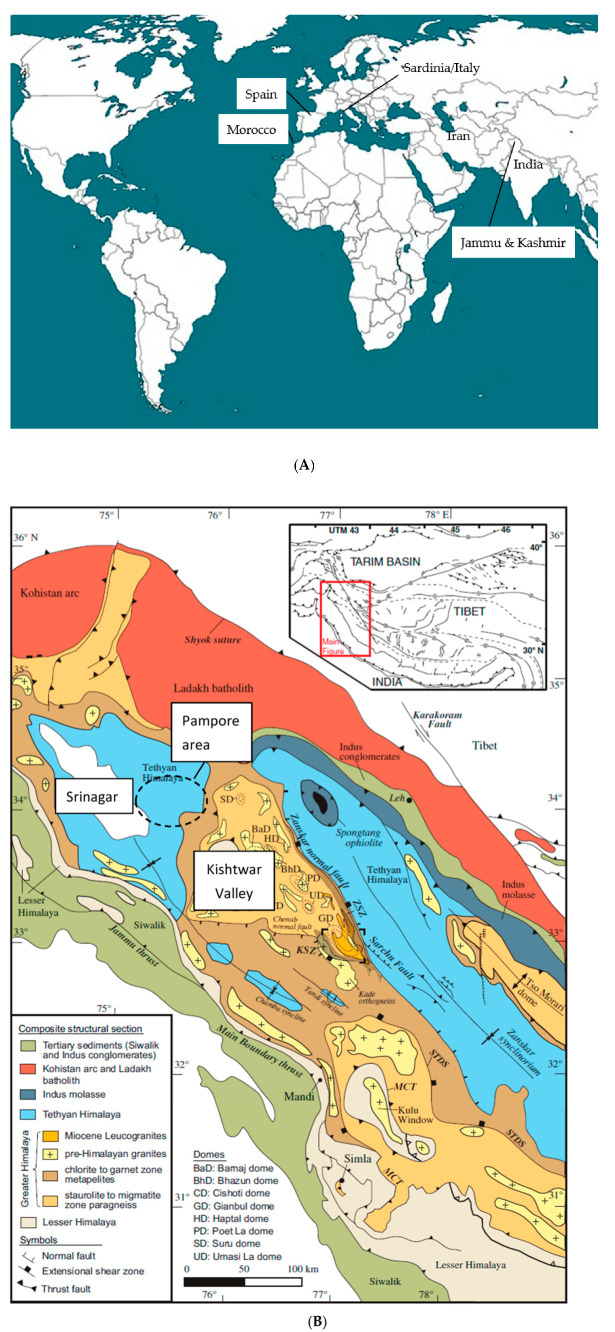
(**A**) Countries of saffron samples origin. The province of Kashmir and Jammu is the northernmost one of India, with Kashmir as the northern part and Jammu the southern part of the province. The Kishtwar Valley is situated in the northeast of Jammu. (**B**) Geological map of Jammu and Kashmir after Horton et al. (2014) [41]. Slightly southeast of Srinagar (capital of Kashmir) is the Pampore area (dashed circle), where most of the Kashmir saffron is grown, within the Tethyan Himalaya area consisting of marine impure carbonates and clastica and recent alluvium. The Kishtwar Valley is geologically situated within an area of granites and gneiss.

In all, 27 saffron samples were collected in different countries and regions, specifically: 9 from Morocco (see Table 1), 13 from India, 3 from Spain (La Mancha region), 1 from Iran, and 1 from Sardinia/Italy (Figure 1A,B). All but one of the Indian samples originated from Kashmir, whereas the one other sample came from the Kishtwar Valley in Jammu Province (Figure 1B). The Moroccan samples were collected by one of the authors (M. Lage). The Kashmir saffron samples were bought by M. Horacek in Kashmir from reliable retailers, the Kishtwar saffron sample was collected by J. Vakhlu, and the other samples were provided by colleagues from the respective countries. Each sample consisted of at least one gram of saffron filaments. The dry samples were stored in either small paper bags or small plastic tubes with tightly sealed caps at room temperature.

### 2.1. Sample Preparation

The samples were homogenized in an agate mortar. A total of 320 mg of saffron was digested with 3 mL of HNO_3_ acid (68%, optima) and 1 mL H_2_O_2_ 31%, after a pre-digestion step (with 3 mL HNO_3_ overnight at room temperature, ca. 22 °C), by using a High Pressure Asher as described elsewhere [42]. Samples were digested in triplicate. The obtained extracts were stored at 4 °C until analysis.

### 2.2. Isotopic Analyses

Approximately 3.5 mL of the digested extract was evaporated until dryness in a hot block at 95 °C. After evaporation, the sample was diluted in 4 mL of HNO_3_ 3M and loaded in a column containing 0.16 g of SR-B50-S resin in order to separate Sr from sample’s matrix. The Sr specific isolation procedure from the matrix was based on the method already detailed by Martin et al. (2013) [43], after obtention of the elution profile of Sr in the saffron matrix. In brief, Sr was eluted by the addition of 10 mL of H_2_O, once the matrix was removed with 8 mL HNO_3_ 3M. The Sr-containing fractions were diluted in HNO_3_ 2% (*v*/*v*) before MC-ICP-MS (Multicollector Inductively Coupled Plasma Mass Spectrometry) analyses.

The analysis was carried out on a “Nu Plasma” MC-ICPMS instrument (Nu Instruments, UK), at Pau University, France, as described in Cellier et al. (2021) [29]. Table S1 shows the instrumental parameters. The isotopically certified standard NIST SRM 987 (pure SrCO_3_, NIST, Gaitherburg, MD, USA, certified for its isotopic composition with a certified value for ^87^Sr/^86^Sr of 0.710255 ± 0.00023) was used as both bracketing standard and quality control. Mass bias and interference correction were carried out as described in Ehrlich et al. (2001) [44]. All samples were measured in triplicate. Standard deviation was usually better than 0.00007.

### 2.3. Multi-Elemental Analyses

Approximately 0.15 mL of the digested sample was diluted in 2% HNO_3_ (*v*/*v*) and among other element isotopes ^85^Rb and ^86^Sr were monitored. In addition, an SRM, in duplicate, and a CRM (Certified Reference Material), in triplicate, both of vegetal origin, were processed. These materials were SRM 1570a Spinach Leaves and CRM No. 23 Tea Leaves II, certified on Sr (55.54 ± 0.5 and 3.93 ± 0.25 mg/kg, respectively) and Rb (12.7 ± 1.6 and 17 (the latter with no certified or reference value) mg/kg, respectively), which were also digested, and the measurements used for the validation of this crucial step. The concentration of Sr was determined by ICP MS in the saffron samples, the SRM and CRM. The obtained value for the 28 saffron samples with different geographical origins was between 1.82 and 16.29 mg/kg. The results were validated by the match of the certified and obtained Sr concentrations in the vegetal SRM and CRM, 50.46 and 4.14 mg/kg, respectively, and Rb concentrations of 12.96 and 16.79 mg/kg, respectively. The limit of quantification of Sr and Rb by ICP MS was usually below 0.05 mg/kg.

The instrument used was a NexION 300x (PerkinElmer Inc., Paris, France) equipped with a universal cell to remove potential interferences, which may have an influence on achieving accurate measurements of the elements. The aforementioned cell can be operated in two different modes, depending on the principles of interference removal: collision or reaction mode. The cell can also be simply turned off when no interference removal is intended to be performed, which is called standard mode. In this work, the collision cell technology (CCT) was used in order to achieve an efficient removal of polyatomic interferences by kinetic energy discrimination (KED) by means of helium as the non-reactive gas. The general operating parameters used for the analysis are shown in Table S2.

### 2.4. Statistical Evaluation

For statistical evaluation the software environment R (Vienna) was used to perform a Kruskal–Wallis Test. The coefficient of determination (R^2^) was calculated using Excel.

## 3. Results

**Figure 2:**
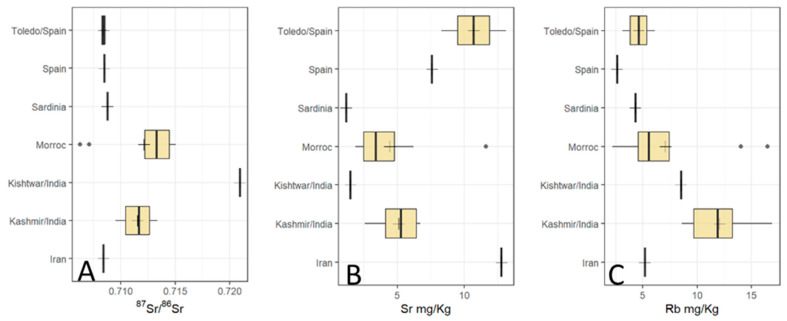
Boxplots of ^87^Sr/^86^Sr, Sr and Rb concentrations with respect to geographic origin. Group centroids are marked by a cross, outliers are shown as dots. (**A**) ^87^Sr/^86^Sr results. Note the narrow range of Spanish, Sardinian (Italy), and Iranian saffron samples within the area of marine ^87^Sr/^86^Sr values. (**B**) Sr concentrations, (**C**) Rb concentrations.

**Figure 3:**
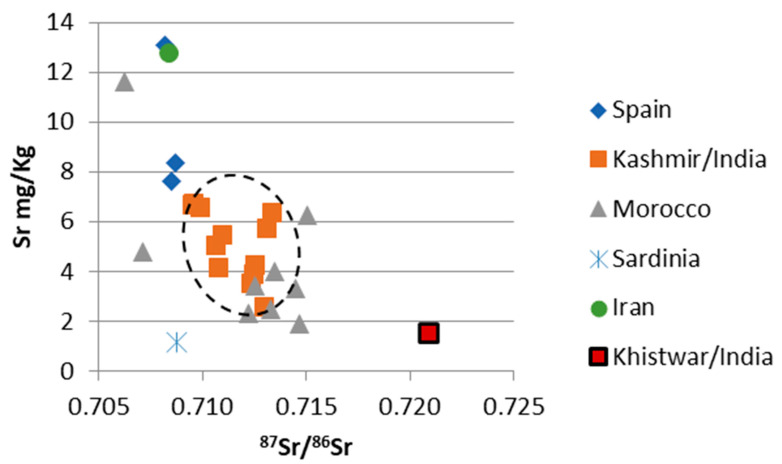
^87^Sr/^86^Sr ratios of the saffron samples versus Sr concentration. Note the difference in pattern between the Kishtwar/India sample and the samples from Kashmir/India. The dashed circle indicates the Kashmir saffron point cloud. For details refer to text.

**Figure 4:**
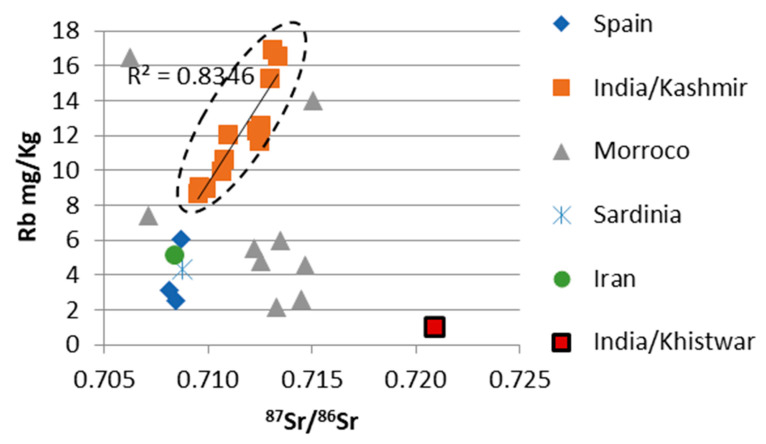
^87^Sr/^86^Sr ratios of the saffron samples versus Rb concentration. The black line denotes positive correlation of ^87^Sr/^86^Sr and Rb concentration for Kashmir/Indian samples. R^2^ gives the coefficient of determination for the Kashmir saffron samples. The dashed circle indicates the Kashmir saffron point cloud.

**Figure 5:**
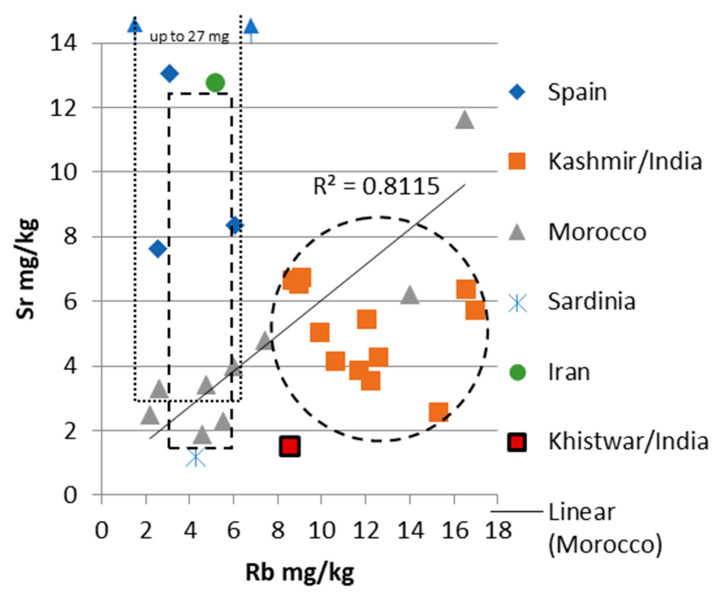
Rb versus Sr concentrations. The black line marks the positive correlation between these two parameters of the Moroccan samples. The dashed circle indicates the Kashmir saffron point cloud. R^2^ gives the coefficient of determination for the Moroccan samples. Squares show strontium and rubidium ranges for Iranian and Spanish saffron from Wakefield et al. (2019). The dashed square is the range of Spanish saffron, the dotted square delineates the range of Iranian saffron values. Blue arrows indicate that the strontium content values of Iranian saffron samples range up to 27 mg/kg, according to Wakefield et al. (2019). For details see text.

A summary of the results is found in Table 2 and in Figure 2, Figure 3, Figure 4 and Figure 5.

**Table 2:** Results of ^87^Sr/^86^Sr, Sr- and Rb-concentrations.

Sample Nr.	Geogr. Origin	^87^Sr/^86^Sr	Sr mg/kg	Rb mg/kg
1	Spain	0.708487	7.6	2.6
2	Toledo/Spain	0.708173	13.1	3.1
3	Toledo/Spain	0.708691	8.3	6.1
4	Kashmir/India	0.712328	3.5	12.2
5	Kashmir/India	0.713128	5.7	16.9
6	Kashmir/India	0.712507	3.9	11.7
7	Kashmir/India	0.713386	6.4	16.6
8	Kashmir/India	0.712517	4.3	12.6
9	Kashmir/India	0.710770	4.2	10.6
10	Kashmir/India	0.710989	5.5	12.1
11	Kashmir/India	0.710686	5.0	9.9
12	Kashmir/India	0.712976	2.6	15.3
13	Kashmir/India	0.709522	6.7	8.6
14	Kashmir/India	0.709904	6.6	9.0
15	Kashmir/India	0.709597	6.7	9.1
16	Kishtwar/India	0.720912	1.5	8.5
17	Morocco	0.713465	4.0	6.0
18	Morocco	0.712228	2.3	5.5
19	Morocco	0.714703	1.9	4.6
20	Morocco	0.713306	2.5	2.2
21	Morocco	0.712514	3.4	4.8
22	Morocco	0.706269	11.6	16.5
23	Morocco	0.715033	6.2	14.0
24	Morocco	0.707133	4.8	7.4
25	Morocco	0.714466	3.3	2.6
26	Sardinia/Italy	0.708791	1.2	4.3
27	Iran	0.708412	12.8	5.2

### 3.1. Rb Concentrations

The Rb concentrations of the saffron samples from Morocco ranged from 2.2 to 16.5 mg/kg with an average of 7.1 mg/kg, the samples from India varied from 8.5 to 16.9 mg/kg with the average of 11.8 mg/kg (Kashmiri samples varied from 8.6 to 16.9 mg/kg with an average of 12.0 mg/kg, and the Kishtwar sample had a Rb content of 8.5 mg/kg), the saffron samples from Spain ranged from 2.6 to 6.1 mg/kg, averaging 3.9 mg/kg, and the Iranian sample and the sample from Sardinia/Italy possessed 5.2 and 4.3 mg/kg, respectively.

### 3.2. Sr Concentrations

The Sr concentrations of the saffron samples from Morocco ranged from 1.9 to 11.6 mg/kg with an average of 4.4 mg/kg, the samples from India varied from 1.5 to 6.7 mg/kg with the average of 4.8 mg/kg (Kashmiri samples varied from 2.6 to 6.7 mg/kg with an average of 5.1 mg/kg, and the Kishtwar sample had a Sr content of 1.5 mg/kg). The saffron samples from Spain ranged from 7.6 to 13.1 mg/kg, averaging 9.7 mg/kg, and the Iranian sample and the sample from Sardinia/Italy possessed 12.8 and 1.2 mg/kg, respectively.

### 3.3. ^87^Sr/^86^Sr Ratios

The Moroccan saffron samples possessed ^87^Sr/^86^Sr values ranging from 0.7063 to 0.7150 with an average of 0.7121. The Indian samples varied from 0.7095 to 0.7230, average: 0.7122. Looking at the two Indian production areas separately, the Kashmiri samples ranged from 0.7095 to 0.7134, average: 0.7115, and the Kishtwar saffron sample had a ^87^Sr/^86^Sr value of 0.7209. The Spanish saffron samples ranged from 0.7082 to 0.7087, with an average of 0.7085. The saffron sample from Iran and the sample from Sardinia/Italy showed ^87^Sr/^86^Sr values of 0.7084 and 0.7088, respectively.

### 3.4. Statistical Evaluations

Statistical evaluation identified one Moroccan saffron sample as an outlier for all three parameters investigated (Figure 2). Furthermore, it also identified the Indian Kishtwar saffron sample as an outlier with respect to ^87^Sr/^86^Sr. Significant differences were identified by the Kruskal–Wallis Test for ^87^Sr/^86^Sr, Sr and Rb concentrations (Table 3A–C). However, many of the groups only consisted of one or just a few samples; thus, these results have to be judged accordingly with caution. Positive correlations were identified (by calculating the coefficient of determination) for Rb concentration and ^87^Sr/^86^Sr in the Kashmir/India samples (R^2^ = 0.8346), and for Sr and Rb concentrations in the Moroccan samples (R^2^ = 0.8115).

## 4. Discussion

For land plants (including all materials and tissues from plants) the relevant parameter is the soil and the underlying geology, especially the type of bedrock (Horacek (2022) and references therein). Marine carbonates vary in their ^87^Sr/^86^Sr values within a small and well-defined range (within 0.7068–0.7092, e.g., [33], and references therein), and the results of the samples from Spain, Iran, Sardinia/Italy, and one Moroccan saffron sample lay within this interval, with variable Sr concentrations within the trend of marine carbonates (varying Sr content, narrow range of ^87^Sr/^86^Sr). The Kashmir/India samples were beyond the range of marine carbonates, evidencing the significant/dominant influence of siliciclastics. In Figure 3, Figure 4 and Figure 5 a black dashed circle outlines the range of the Kashmir saffron samples, indicating a well-confined range for these samples. A positive correlation of ^87^Sr/^86^Sr with Rb content (R^2^ = 0.8346) is noted.

The Rb and Sr contents of the Moroccan samples showed a positive correlation (R^2^ = 0.8115); however, we currently do not have an explanation for that. As saffron of no other origin followed this trend, and as Morocco has a diverse bedrock geology, it might be that there is no single cause for this pattern. The Kashmir/India samples possessed a distinct pattern and only slightly overlapped with a few Moroccan samples. In addition, the Kishtwar sample had a distinctive signal (with respect to ^87^Sr/^86^Sr and Rb and Sr concentrations) and was separated from other saffron samples. The Spanish and Iranian samples showed similar rubidium values (ca. 1.5 to 6.5 ppm) and a stronger variation in Sr concentration between 1 and 13 ppm. Our Spanish and Iranian results were in good agreement with Wakefield et al. (2019) [16] (even though these data might need to be regarded cautiously (see above)). Wakefield et al. [16] reported a slightly smaller range in Rb and Sr for the Spanish saffron than our data suggested, and a much broader range toward higher Sr values. Potentially, elevated Sr concentrations might be a useful indicator for Iranian saffron, even though not all of the Iranian saffron will be identified in this way.

The Moroccan samples showed a large variation in ^87^Sr/^86^Sr isotopes, ranging from below the marine strontium isotope interval to values above 0.715 and thus within the massively siliciclastically influenced range. The sample with the lowest value (0.7063, below the marine interval) evidenced the origin from a basalt bedrock ([33] and references therein), and the sample within the marine strontium isotope range might evidence the origin from a region with carbonate bedrock; however, an origin from a basalt bedrock with a very minor siliciclastic influence might also be possible. The other Moroccan samples possessing ^87^Sr/^86^Sr values beyond the marine range evidenced a dominant siliciclastic influence exceeding the one on the Kashmiri samples, with a small overlap of these two groups between 0.71228 and 0.71338. The highest values also exceeded the range of “continental volcanics”, as shown in Horacek (2022) [33] and references therein. A positive correlation of Sr and Rb content was noted for the Moroccan samples, which is probably founded on the bedrock mineralogy and petrology (with the highest element concentrations in the basaltic bedrock sample). As the ^87^Sr/^86^Sr values also varied strongly within one locality (Taliouine), at least there a very heterogenous geological situation can be assumed also at small scale. The Kishtwar saffron sample showed a very elevated ^87^Sr/^86^Sr value of almost 0.720, indicating a bedrock of (old) granite or gneiss [33], and references therein, as it was present in the Kishtwar Valley area (Figure 1B). Differentiation of geographic origin by ^87^Sr/^86^Sr ratio of the investigated samples is possible for the Moroccan and Kashmiri saffron samples and the Spanish and the Iranian samples. However, as Spain and Iran are both large countries, each possessing a very diverse and heterogenous geology, it needs to be verified if all saffron produced in these countries stems from areas with carbonate bedrock. Still, the ^87^Sr/^86^Sr value reported for Spanish apricots was also within the range observed for the Spanish saffron [40]. No differentiation between the Spanish and the Iranian samples or the Kashmiri and Moroccan saffron was possible judging solely by ^87^Sr/^86^Sr. However, with respect to the latter two countries of origin, a differentiation between Kashmir and Moroccan saffron might be possible using ^87^Sr/^86^Sr, Sr and Rb concentrations. An excellent separation was achieved between saffron from the Kashmir and Kishtwar regions, as the respective differences in bedrock geology resulted in distinctively different ^87^Sr/^86^Sr signals for saffron from these two regions—even though, up to now we only have one result from the Kishtwar Valley. As both regions also seem to be well confined (geographically, with respect to environmental conditions), and the underlying bedrock geology seems to be homogenous at and differing between these sites (Figure 1B), no overlap is to be expected. However, this assumption needs to be tested and verified.

As the results of the Kashmir and Kishtwar saffron samples demonstrated, the analysis of the ^87^Sr/^86^Sr value can be an excellent tool for differentiation of commodities from different geographic regions, given that they possess respective differences and homogeneity in their bedrock geology. Furthermore, a differentiation of geographic origin by ^87^Sr/^86^Sr might be achieved for certain regions by combining the ^87^Sr/^86^Sr ratio with Sr and Rb elemental concentrations, as was shown for the Kashmir and Moroccan saffron samples. However, one has to consider that the bedrock geology in Morocco is obviously very heterogenous; thus, we cannot be certain that we obtained a full spectrum of the ^87^Sr/^86^Sr values possible. Potentially, further proxies need to be applied for a complete differentiation of saffron samples from these two areas. Thus, as already mentioned, the power and potential of the ^87^Sr/^86^Sr analysis depends on the exact question one wants to address—and the respective homogenous conditions of the bedrock of the regions to be differentiated.

## 5. Conclusions

The ^87^Sr/^86^Sr ratio of saffron samples from different regions and countries of origin was analyzed and used for differentiation. An excellent separation was achieved between the saffron samples from the Kashmir and Kishtwar regions, owing to the respective differences in bedrock geology, even though at present this separation was documented for only one sample coming from the Kishtwar Valley/India. A good differentiation between the Kashmir and Moroccan saffron samples investigated was possible by combining the ^87^Sr/^86^Sr value and Sr and Rb concentrations. The investigated saffron samples from Morocco and Kashmir were completely differentiated from the investigated (few) samples from Spain and Iran, as the samples from the latter two countries exclusively showed ^87^Sr/^86^Sr values within the range of marine carbonate bedrock. The Kashmir samples all lay beyond the marine ^87^Sr/^86^Sr range, whereas the Moroccan samples investigated lay beyond or below and one Moroccan sample was within the range of marine carbonate ^87^Sr/^86^Sr, but this sample could be differentiated by the Sr and Rb contents. However, both Spain and Iran are big countries with a very heterogenous geology; thus, it might be expected that saffron samples from these two countries would possess ^87^Sr/^86^Sr values exceeding the ones measured in our study, even though the production of saffron in Spain is restricted to a small region. Therefore, the good differentiation observed in this study might disappear when analyzing further samples from these two countries. Our study confirmed the excellent differentiation of saffron from Kashmir and Kishtwar (even though based on only one sample from Kishtwar so far) because of the seemingly homogeneous geological situation in the saffron-growing area in Kashmir (Pampore region) and the distinctively differing geology in Kishtwar. Furthermore, the Moroccan and Kashmir samples could be differentiated from each other and from the investigated samples of other geographic origins applying ^87^Sr/^86^Sr analysis and Sr and Rb content investigations. However, this claim needs to be checked and confirmed by analysis of further saffron samples.

## Figures and Tables

**Table 1:** Moroccan saffron samples.

Sample Nr. *	Sample Description	Origin	Altitude	Vintage
**17**	Sample 1; Moroccan Saffron	Taliouine-mixture (different farmers)	-	2011
**18**	Sample 2; Moroccan Saffron	Rabat	56	2012
**19**	Sample 3; Moroccan Saffron	Marrakech	1500	2011
**20**	Sample 5; Moroccan Saffron	Taznakht	1800	2011
**21**	Sample 6; Moroccan Saffron	Taliouine	1404	2011
**22**	Sample 7; Moroccan Saffron	Taliouine	1792	2011
**23**	Sample 8; Moroccan Saffron	Taliouine	1597	2011
**24**	Sample 9; Moroccan Saffron	Commercial	-	2011
**25**	Sample 10; Moroccan Saffron	Taznakht	1604	2011

* sample numbers refer to Table 2.

**Table 3:** Kruskal–Wallis Test: Identification of significant differences. Different letters identify significant differences.

**A: ^87^Sr/^86^Sr.**		
	**^87^Sr/^86^Sr**	
Kishtwar/India	27.000	a
Morocco	17.000	ab
Kashmir/India	14.417	abc
Sardinia/Italy	7.000	abc
Spain	5.000	bc
Toledo/Spain	4.500	c
Iran	4.000	c
**B: Sr concentrations**		
	Sr mg/kg	
Iran	26.000	a
Toledo/Spain	25.500	a
Spain	23.000	ab
Kashmir/India	15.000	ab
Morocco	10.556	bc
Kishtwar/India	2.000	c
Sardinia/Italy	1.00000	c
**C: Rb concentrations**		
	Rb mg/kg	
Kashmir/India	19.917	a
Kishtwar/India	13.000	ab
Morocco	10.667	b
Iran	8.000	b
Toledo/Spain	7.500	b
Sardinia/Italy	5.000	b
Spain	2.000	b

## Data Availability

The datasets generated for this study are included in this article.

## Supplementary Materials

The following supporting information can be downloaded at: https://www.mdpi.com/article/10.3390/foods12152830/s1. Table S1: Instrumental operating conditions for MC-ICP MS. Table S2: Instrumental operating conditions of the PerkinElmer NexION 300X ICP-MS.
