# Supplementary material for: Exploring the Potential of 87Sr/86Sr Isotope Ratio with Strontium and Rubidium Levels to Assess the Geographic Origin of Saffron
Source: Foods. 2023 Jul 26;12(15):2830. doi: 10.3390/foods12152830 (PMC10416822; doi:10.3390/foods12152830)
Supplement: Supplementary file 1 [file foods-12-02830-s001.zip › foods-2373552-supplementary.pdf]

Table S1. Instrumental operating conditions for MC-ICP MS.

INSTRUMENTAL PARAMETERS	
**RF power (W)**	1300
**Acceleration voltage (V)**	6000
**Gas parameters (Ar)**	
Plasma gas flow rate (L.min^{-1})	13
Auxiliary gas flow rate (L.min^{-1})	0,8
**Instrument resolution**	Low resolution (~ 400)
**Aquisition parameters**	
Number of blocks	3
Number of measurments/block	20
Integration time (s)	10
Total acquisition time (s)	600
**Wash time (s)**	240
**Uptake time (s)**	240
**Nebulizer**	200µL/min micro-concentric
**Spray chamber**	Cinnabar cyclonic
**Interface cones**	Nickel Wet plasma cone (Type A)

Table S2. Instrumental operating conditions of the PerkinElmer NexION 300X ICP-MS.

Component/Parameter	Type/Value/Mode
Nebulizer	Meinhard® glass microcentric
Spray chamber	Glass cyclonic
Triple Cone Interface Material	Nickel/Aluminum
Plasma Gas Flow	15.0 L/min
Auxiliary Gas Flow	1.2 L/min
Nebulizer Gas Flow	0.96 L/min
Sample uptake rate	250 µL/min
RF Power	1100 W
Dwell time per amu	50 ms
Replicates per sample	3
Sweeps per reading	50
Mode of operation	Standard and collision (Helium at 3.0 mL/min)
$^{156}CeO^{+}/^{140}Ce^{+}$	0.018
$^{70}Ce^{++}/^{140}Ce^{+}$	0.010
